# Development of the MOOSY4 eNose IoT for Sulphur-Based VOC Water Pollution Detection

**DOI:** 10.3390/s17081917

**Published:** 2017-08-20

**Authors:** Enric Climent, Jose Pelegri-Sebastia, Tomas Sogorb, J. B. Talens, Jose Chilo

**Affiliations:** 1 Sensors and Magnetism Group, Institut de Recerca per a la Gestió Integrada de Zones Costaneres, Campus de Gandia, Universitat Politècnica de València, 46730 Grao de Gandia, Spain; enclimar@alumni.upv.es (E.C.); tsogorb@eln.upv.es (T.S.); juatafe@epsg.upv.es (J.B.T.); 2 Department of Electronics and Physics, University of Gävle, SE-80176 Gävle, Sweden; jose.chilo@hig.se

**Keywords:** electronic nose, water quality, embedded, WEKA, ANN, MOOSY4

## Abstract

In this paper, we describe a new low-cost and portable electronic nose instrument, the Multisensory Odor Olfactory System MOOSY4. This prototype is based on only four metal oxide semiconductor (MOS) gas sensors suitable for IoT technology. The system architecture consists of four stages: data acquisition, data storage, data processing, and user interfacing. The designed eNose was tested with experiment for detection of volatile components in water pollution, as a dimethyl disulphide or dimethyl diselenide or sulphur. Therefore, the results provide evidence that odor information can be recognized with around 86% efficiency, detecting smells unwanted in the water and improving the quality control in bottled water factories.

## 1. Introduction

At present, the number of sensor devices connected to the Internet of Things (IoT), referring to different object types of embedded devices with internet connection (low-power and low-cost devices), is rapidly growing to support new and more daring applications thanwas predicted many years ago [1]. The IoT currently offers development of a broad class of applications in various areas: smart environments (smart homes, cities, office and industrial environments), transportation and logistics, healthcare, surveillance, and other environments [2]. Industry 4.0 comprises the connection of intelligent sensors and devices to create a complete digital value chain, the Industrial Internet of Things. Nowadays, the integration of gas sensors is apriority in Industy 4.0; recently, some companies have been working in thearea ofgas sensors and IoT, such as Spec Sensors, Sensirion, SGX Sensortech or Rubix.

Current research on sensors and the IoT shows that odor sensors are onlystarting to be integrated in the same range as other types of sensors. With regard toenvironmental sensors, these are mainly used to measure temperature, humidity and displacement [3], as well asthe Smart Citizen platform [4], whichincludes CO and NO_2_ gas sensors for automotive applications. Other sensors are commonly used for healthcare applications to record Electrocardiography (ECG), Electroencephalogram (EEG) and Electromyogram (EMG) data [5]. For industrial applications, there are no frequently-used gas sensors [6,7].

An eNose instrument is able to simulate the human nose [8], replicating the four functions of the sense of smell: detection, recording, memory search and identification. The detection and recording functions are simulated by the use of an array of gas sensors, a signal conditioning electronics, an Analogue to Digital Converter (ADC) [9] and, lastly, a processor unit (such as a microcontroller, PC, etc.) with software for feature extraction and classification [10]. The eNose with a sensor array, with each sensor with a different response to gases, adopt cross-sensitivity gas sensors. Therefore, there are a greater number of different types of sensors in the array, and these have a different level of response foreach volatile compound in the sample mixture. Then, using mathematical approaches may permit the extraction of the desired volatile compound response from the fusion of responses from the multiple sensors [11].

Different types of eNose have been developed extensively by researchers in recent years for various purposes, and [12] summarizes electronic-nose technologies and applications with high benefit to man, such as detection of or discrimination between different substances, including liquors [13,14], toxic gases [15], tobacco [16] or smoke and non-fire particles [17], and for the agriculture and forestry industries [18], for ethanol sensing [19] or for environmental monitoring [20] and healthcare applications [21]. Some research has been conducted to find rapid and simple ways to achieve early detection of potential water contamination. It has been proved that eNose systems can be successfully utilized as a method fordetecting the presence of some substances in water like geosmin [22], cyanobacteria [23], Escherichia coli, or enterobacteraerogenes [24], and for the detection of chemical contamination [25]. In [26], it was concluded that bottled water that smells different fromnormal, e.g., like rotten eggs, wet cloth, butane or rubber, has the presence of volatile dimethyl selenides or dimethyl sulphides in different concentrations. Moreover, there are some IoT applications for the detection of evaporatedhazardous materials with a gas sensor network [27,28,29].

Traditional eNoses with large amounts of sensors, from 8 to 32 or more, raisedifficulties in terms of integrating themin the embedded system for IoT applications (oriented to low-power and low-cost applications), because the computingand electrical power consumption demanded bythe system is high. Furthermore, feature parameter extraction and classification plays an important role in the performance of electronic noses. Therefore, it is important to optimize the sensor array, the extracted feature subset and classifications algorithms on eNose systems to be integrated widely in IoT.

In this paper, we present an electronic nose with only four gas sensors for sulphur-based VOC water pollution detection. The sensors are chosen from the low-power Figaro family TGS26; this feature makes the electronic nose suitable for the IoT with mains. Applications and eNose are integrated together into a smooth and coherent system. eNose users can run an experiment attheir own place, and the results can be easily visualized by a comprehensible user interface. The results are stored in the user’s profile inthe cloud, and other users can access the results.

The rest of this paper is organized as follows: Section 2 introduces the materials and methods in detail of the eNose system developed. Section 3 presents the results and discussion ofthe design and the experiment for detection of volatile components in water pollution. Finally, Section 4 concludes the paper.

## 2. Materials and Methods

The multi-sensory odor olfactory system used to choose the best sensors has 32 metal oxide semiconductors (MOS) sensors (MOOSY32). In MOOSY32, TGS2610-c00 and TGS2610-d00 areused for LP gas, TGS2611 for methane, TGS2620 for alcohol, solvents and vapor, with an accuracy of 500–10,000 ppm and TGS2600 for air contaminants with an accuracy of 1–30 ppm. The last one, TGS2600, has a poor accuracy in comparison to the others, but it can detect a wide range of air contaminants.

It is very common to use electronic chemical sensors with partial or no specificity. This situation purports to have different sensitivities against different substances. This study has tried to identify the sensors that offer greatest signals, thus reducing the dimensions and the cost of the system. This approach to minimizing eNose has tried to reduce the current sophisticated desktop systems, such as MOOSY32 with 32 gas sensors for glucose detection in humans [30], toa simple embedded system that can be produced at low cost and is easy to use. The MOOSY4 eNose for IoT was designed on the basis of this eNose.

**Figure 1:**
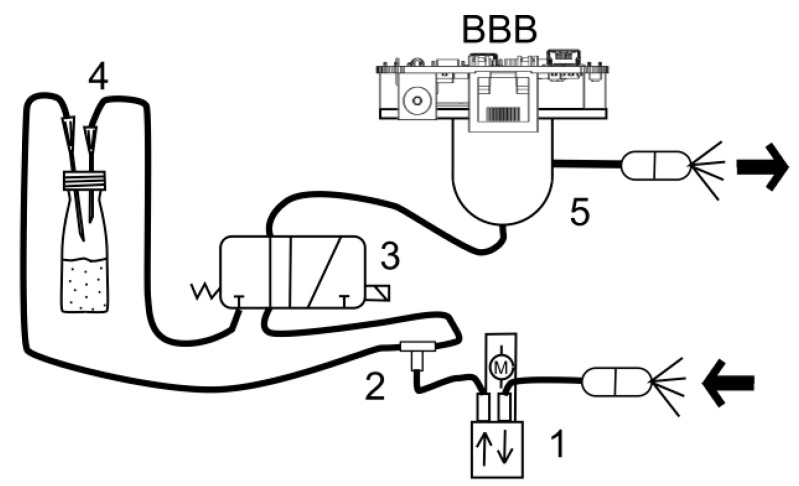
Architecture of the MOOSY4 eNose system: Pump (**1**), T connector (**2**), 3/2 valve (**3**), substance under test (**4**), chamber with sensors (**5**).

The functional principles and the architecture of the MOOSY4 eNose can be easily summarized in Figure 1. Itis mainly composed of a pneumatic system, a gas sensor array and a Beaglebone Black (BBB) for control, data collection, signal processing, pattern recognition and connection to the cloud. Clean air from the air pump (1) is redirected using a T-connector (2) and anelectronic valve (3) via the substance to be smelled (4), or directly to chamber (5) with gas sensors for cleaning. Gas sensors are inserted in a closed chamber with air inlet and outlet. The pump and valve arecontrolled directly from the BBB, where parameters, like testing and cleaning time, can be set depending on different applications.

A Beaglebone Black with 24-bit ADC and internal memory of 4GB multimedia card (eMMC) is used. According to the characteristics of the sensors and their response sensitivity, asa first step we check some samples (Table 1) in the MOOSY32, and subsequently selected the sensors with the best response signal. Four Figaro gas sensors were carefully selected: TGS2600, TGS2610, TGS2611 and TGS2620. The sensitivity range of each of the four Figaro gas sensor types in terms of VOC sensitivity to specific gas types is givenin Table 2. The gas sensor array wasused to measure the cross-sensitivity of a variety of gases, and by using appropriate pattern recognition methods, the substances wereclassified.

### 2.1. Data Acquisition

**Figure 2:**
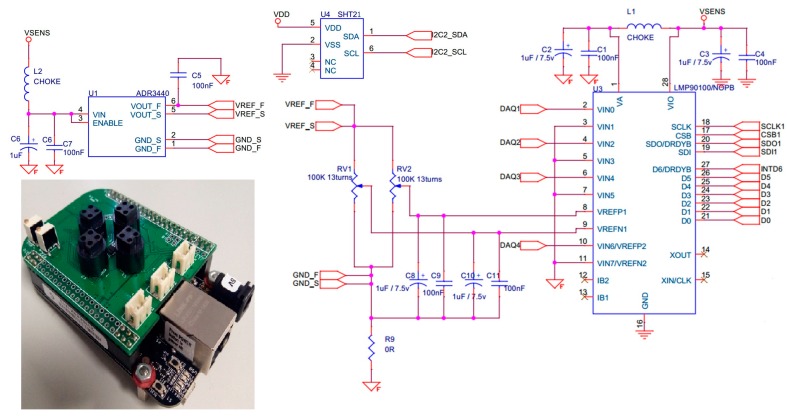
Data acquisition expansion board.

We built an expansion board with connections for gas sensors, temperature and humidity sensor and the LMP90100 AD converter from Texas Instruments was selected. The ADC has 24-bit resolution, low-noise programmable gain and an SPI serial interface. The SHT21Q2009 temperature and humidity sensor from Sensirion was used. Additionally, a voltage reference was necessary, for which the ADR3440 from Analog Devices was selected. Figure 2 shows the scheme and the photo of the expansion board.

### 2.2. Experiment Description and Data Processing

A 10 mL screw-top vial of transparent glass from Scharlab was used for the sample. The air was introduced using two kinds of needles, and the experiments were performed by preheating samples to 40 °C. The system cleanedthe chamber for 20 s, and thenwaited for 15 s before opening the duct with gas from the sample. The sample gas was passedfor 135 s. The airflow of the sample was closed, following which a new cycle of measurement was started.

For each experiment, account was taken of the current valuesforhumidity and temperature. In our case, we used algorithms to compensate for deviations, using a mathematical model to obtain the equations from the graphics of the data sheet of manufacturer [31]. Three equations wereappliedto the different types of sensors. Equation (1) was used for TGS2600, Equation (2) for TGS2610 and TGS2611 and Equation (3) for TGS2620:

*R_TGS2600_* = *R_0_* × e^A^,

where A is

A = 1.34686 − 0.048203·*T* + 0.000409051·*T*^2^ − 0.0100806·*RH* + 0.000102265·*T*·*RH*, (1)

*R_TGS2610_* = *R_0_* × e^B^,

where B is

B = 1.81406 − 0.0376227·*T* + 0.000190549·*T*^2^ − 0.0029376·*RH* + 0.0000380571·*T*·*RH*, (2)

*R_TGS2620_* = *R_0_* × e^C^,

where C is

C = 0.7115 − 386.210 × 10^−^^6^·*T*^2^ + 4.5665 × 10^−^^6^·*T*^3^ + 6.6688 × 10^−^^3^·*RH* − 151.691 × 10^−^^6^·*RH*^2^ − 754.391 × 10^−^^6^·*T*·*RH* + 3.1555 × 10^−^^6^·*T*^2^*RH* + 5.9011 × 10^−^^6^·*TRH*^2^, (3)

where *R* is the resistance of the sensor, *R_0_* is the nominal resistance of the sensor, *T* is the temperature of the chamber, and *RH* is the relative humidity.

#### Data Processing

The feature selection wasperformed through a correlation-based approach: Correlation based Feature Selection (CFS). CFS is a filter algorithm that ranks feature subsets according to a correlation-based evaluation function. The goal of the evaluation function is find subsets that contain features that are highly correlated with the class and uncorrelated with each other. Irrelevant features should be ignored, and redundant features should be screened out [32]. In this work, we used CFS Subset Evaluation with a Greedy Step-Wise Ranker, implemented in WEKA (open source software) [33].

The sensor responses were analyzed usingmultivariable classification analysis with WEKAsoftware, using different algorithms. The algorithms applied were the Nearest Neighbor algorithm (NNA) and Multilayer Perception (MLP). The technique of the ‘Nearest Neighbor’ was used to classify new instances, the similarity function to calculate the similarity between the training instance and the instances of the data set [34,35]. Multilayer Perception (MLP) uses an artificial neuralnetwork that makes it possibleto create a model during a training test thatis able to classify the data [36].

The classification analysis consists in the organization of data in classes, using given class labels to order the objects in the data collection. Classification approaches normally use a training set in whichall objects are already associated with known class labels. The classification algorithms learn from the training set to build a model. The model is used to classify new objects [37]. The artificial neural networks learn from examples through iteration, without requiring a priori knowledge of the relationship among variables under investigation [38].

### 2.3. Modular Platform of the MOOSY4 IoT

**Figure 3:**
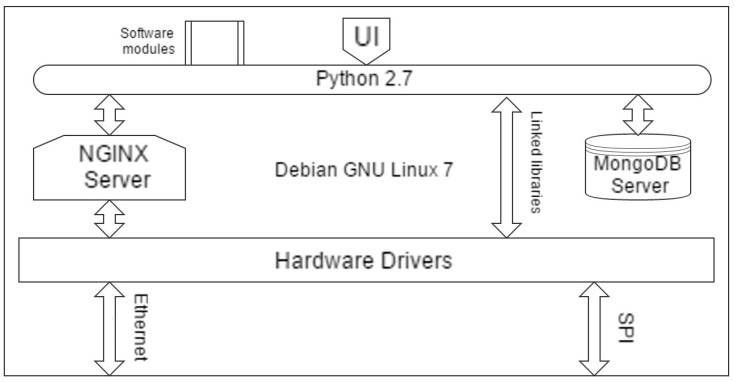
Modular platform of our system.

Figure 3 shows the modular platform of the MOOSY4 system based on Internet of Things technology. To connect with an IP network, the gas sensors use an SPI module for communication with the BBB, and then withthe internet-based IP network. A database server system, such as the MongoDB Server, collects the data of the eNoses. The service provider commands the eNoses to sense data, the eNoses receive the command and process the functions. The default operating system is Debian ARM7.8 and the Python language wasused to program the top. Additionally, we used a NGINX server for web communication, which acted as an intermediary between the server platform and incoming requests.

## 3. Results and Discussion

We tested the MOOSY4 eNose developed in an application for Sulphur-based VOCs in bottled water. Bottled water companies confront major problems controlling water pollution, mainly because of undesirable components release from the ground in connection with heavy rain. Bacteria and other microbes in water can make the water toxic, or make it smell bad. Water contamination is usually tested by growing bacteria in the lab or by gas chromatography or mass spectroscopy. In our work, we used gas sensors to smell water. Sometimes, bottled water with different smells, such as rotten eggs, wet cloth, butane or rubber, showed the presence of volatile dimethyl selenides and dimethyl sulphides, whose concentrations ranged, respectively, from 4 to 20 ng/L and from 1 to 63 ng/L [29].

There are a number of different types of Figaro gas sensors. We very carefully selected four gas sensors forour specific application. Table 1 shows five different water samples based on reference [29], where the compounds that can generate obnoxious smells were analyzed, and which reported the presence of dimethyl sulphides and dimethyl selenides causing odor problems in bottled waters. The water samples used in our analysis were: Sample 1: Water combined with Dimethyl disulphide; Sample 2: water with dimethyl trisulphide; Sample 3: dimethyl diselenide; Sample 4: Water mix with sulphur compounds (water directly extracted from an aquifer that smells like sulphur, but the composition of which is unknown); Sample 5: Commercial water.

**Figure 4:**
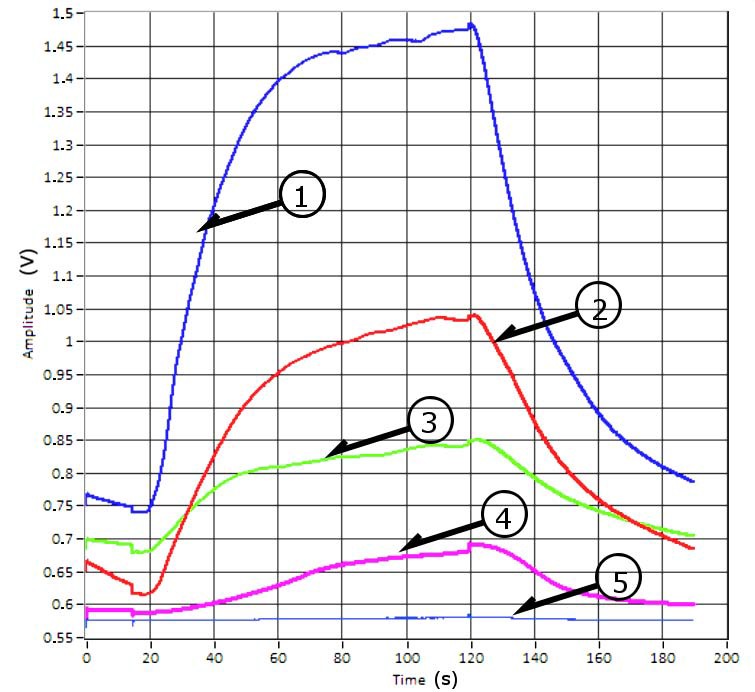
Signals captured with sensor TGS2610-c00from the MOOSY4 eNose. These correspond to samples (1–5) from Table 1.

Figure 4 shows the response of sensor TGS-2610-c00, when exposed to the various water types. 

Two classification algorithms were used in this work: the nearest neighbor algorithm (NNA), which is based on scoring the distances between two instances according to the similarities of its attributes, and the neural network Multilayer Perceptron (MLP), based on the use of an activation function based on the number hidden layers. Experiment results showed that CFS and MLP or NNA quickly identifies the different substances. 

The results are illustrated in the confusion matrix, which represents the accuracy of the solution of the classification problem. It allows the visualization of the performance of an algorithm. Each column of the matrix represents the instances in a predicted class, while each row represents the instances in the actual class. The ideal result is to have all the samples end up on the diagonal cells of the matrix [33].

**Figure 5:**
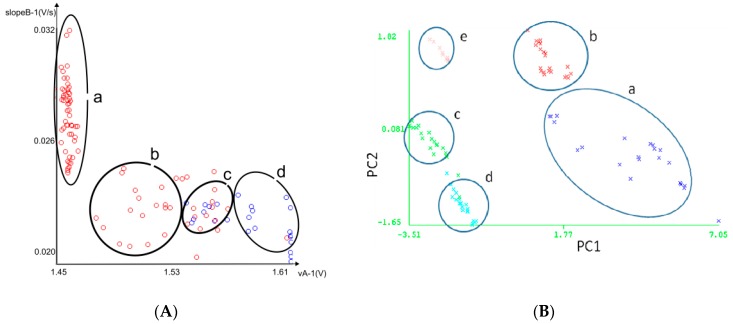
(**A**) Samples processed with MLP. (a) Dimethyl disulphide, dimethyl trisulphide, dimethyl diselenide. (b) Mix of sulphur compounds. (c) Confusion. (d) Commercial water. (**B**) Samples in a PCA graphic: (a) Dimethyl disulphide, (b) dimethyl trisulphide, (c) dimethyl diselenide, (d) Mix of sulphur compounds, (e) Commercial water.

The classification features utilized for this study were: the maximum value of the rising part of the curve (vA), and the rising slope curve (slopeB) about Figure 4. Figure 5A represents the typical graphical response of the WEKA software for the analysis of samples using MLP, for the analysis features slopeB and vA. And Figure 5B represents a PCA graphic.

**Figure 6:**
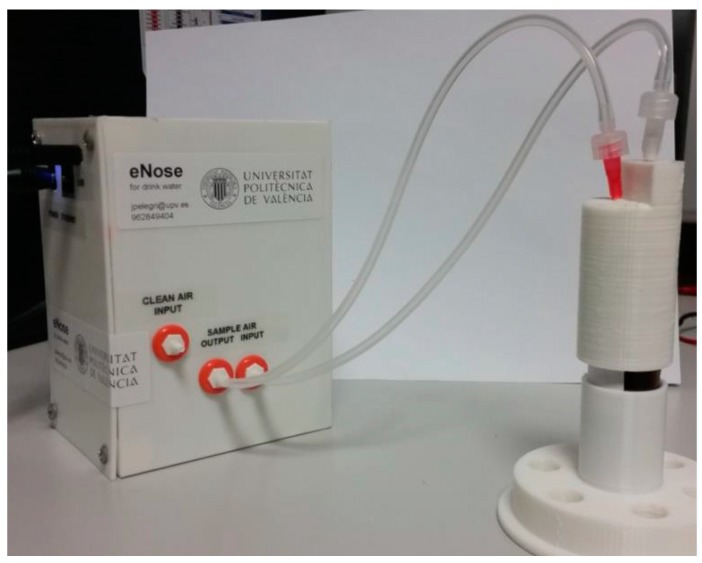
Picture of the low power and low cost MOOSY4 eNose for IoT designed.

For this application, we were interested in the discrimination of commercial water, (d) in Figure 5A, from one of the four contaminant samples in Table 1, (a–c) in Figure 5A, i.e., detecting undesirable smells in the water and improving the quality control in water factories (drinking water from non-drinking water). In addition, Table 3 shows that the eNose system accurately distinguished water contaminants using NNA and MLP methods for classification. Moreover, Figure 6 showsa picture of the low-power (maximum 1.5 W) and low-cost MOOSY4 eNose for IoTthat was designed. The workings inside the box are described by Figure 1.

## 4. Conclusions

In this work, we have developed an electronic nose with a four metal oxide gas sensors and a modular platform based on Internet of Things technology. That can be integrated into IoT Technology with the help of a Beaglebone Black. The four gas sensors are chosen depending on application. The MOOSY4 with the processing method used was able to distinguish water contaminants using commercial Figaro sensors, series TGS-26xx.

The results showed that something changed in the aromatic pattern of water when it containedcontaminants, such a dimethyl sulphides or dimethyl selenides, and that the pattern variation wasdifferent on the different contaminants. The results showed that it is possible to distinguish sulphur-based VOC water pollution in the drinking water with up to 86% accuracy for correct identifications using the features slopeB and vA with the Multilayer Perception classification algorithm.

## Figures and Tables

**Table 1:** Samples to study. The number of replications of each sample type was 4.

Sample	VOC Gases	ppb
1	Dimethyl disulphide (C_2_H_6_S_2_)	100
2	Dimethyl trisulphide (C_2_H_6_S_3_)	100
3	Dimethyl diselenide (C_2_H_6_Se_2_)	100
4	Odor sulphur (unknown)	-
5	Commercial water	-

**Table 2:** Figaro sensor models with features.

Sensors	Model	Target GAS	DetectionRange
1	TGS2600	H_2_ and alcohol (Air contaminants)	1–30 ppm
1	TGS2610-c00	Liquefiedpetroleum gas	500–10,000 ppm
1	TGS2611	Methane	500–10,000 ppm
1	TGS2620	Alcohol, solvents, vapour	500–5000 ppm

**Table 3:** Results of MOOSY4 depending on the algorithm used for 161 instances (*k* = 1) and for the samples of Table 1 in two groups: commercial water vs the others samples.

	Cross Validation 90% (*N* =)		Cross Validation 66% (*N* =)	
Algorithm	Correctly Classified	Incorrectly Classified	Correctly Classified	Incorrectly Classified
NNA	83.23% (134)	16.77% (27)	76.36% (42)	26.64% (13)
MLP	85.71% (138)	14.29% (23)	83.63% (46)	16.36% (9)

## Abbreviations

The following abbreviations are used in this manuscript:

NOSE	Electronic nose.
MOS	Metal oxide semiconductors.
MOOSY4	Multi-sensory odor olfactory system with 4 sensors.
MOOSY32	Multi-sensory odor olfactory system with 32 sensors.
ECG	Electrocardiography
EEG	Electroencephalogram
EMG	Electromyogram
LP gas	Liquefied petroleum gas.
OSI model	Model of architecture for open systems interconnection.
BBB	Beagle bone black.
ARM	Advanced RISC machines.
Weka	Waikato environment for knowledge analysis.
NNA	Nearest neighbour algorithm.
MLP	Multilayer perceptron.
GVA	Valencian government from Spain.
